# Preliminary dosimetric comparison between fixed and rotating source stereotactic radiosurgery systems

**DOI:** 10.1002/acm2.13907

**Published:** 2023-01-19

**Authors:** Emily Draeger, Zhe (Jay) Chen, James E. Hansen, Veronica Chiang, Christopher J. Tien

**Affiliations:** ^1^ Department of Therapeutic Radiology Yale University School of Medicine New Haven Connecticut USA; ^2^ Department of Neurosurgery Yale University School of Medicine New Haven Connecticut USA

**Keywords:** Gamma Knife, rotating source gamma system, stereotactic radiosurgery

## Abstract

**Purpose:**

The Akesis Galaxy RTi (AK) is a novel rotational ^60^Co‐based cranial stereotactic radiosurgery (SRS) system. While similar systems have been compared against the fixed‐source Leksell Gamma Knife (GK) system using stylized phantoms, dosimetric plan quality with realistic anatomy has yet to be characterized for this or any other rotating system versus GK. This study aims to benchmark AK dosimetric performance against GK by retrospectively replanning previously‐treated GK patients at our institution.

**Methods:**

Thirteen patients, previously treated on a GK Icon, were re‐planned on the AK treatment planning system using the same prescription doses and isodoses as the original GK plans. The cohort includes patients treated for brain metastases, schwannomas, pituitary adenomas, trigeminal neuralgias, and arteriovenous malformations. Plans are evaluated with target coverage metrics (D_min_, D_mean_, D_95%_, V_150%_) and dose conformality indices: Radiation Therapy Oncology Group conformity index (CI), selectivity, Paddick CI (PCI), gradient index (GI).

**Results:**

AK plans use fewer shots and larger collimation compared to GK plans, resulting in statistically significant reductions in treatment time (*p* = 0.047) by as much as 88.4 minutes while maintaining comparable target V_100%_. For most metastatic cases, GK produces higher D_min_ (16.0–25.9 vs. 12.5–24.3 Gy, *p* = 0.008) while AK produces higher V_150%_ (0.03–14.92 vs. 0.02–11.59 cc, *p* = 0.028). For non‐metastatic cases, GK provides superior CI (*p* = 0.025) and GI (*p* = 0.044). No statistically significant differences were found in the remaining metrics.

**Conclusion:**

This cohort demonstrates that the AK system is able to achieve largely comparable dosimetric results to GK, typically with shorter treatment times. Further investigation with a larger cohort is underway.

## 1 INTRODUCTION

The Leksell Gamma Knife (GK) stereotactic radiosurgery (SRS) system has been in use since the 1960's,^1^ and much literature has demonstrated the effectiveness of GK‐based SRS.^2^, ^3^, ^4^ Depending on the GK model, the unit contains anywhere from 192 to 201 sources. One of the major downsides of this system is the relatively large number of ^60^Co sources, as it can cause security concerns and higher costs for source replacement. In turn, this may make GK inaccessible to clinics with limited budgets and/or lower‐income regions. Further, mainly due to the large number of sources, source loading (or reloading) typically results in several weeks of downtime, disrupting the SRS program and patient treatment.

To address issues stemming from the large number of sources required, investigators in Asia and within the US have been exploring novel rotating gamma‐source systems. The rotating delivery approach is based on the premise of rotating a tray containing ∼50 high‐source‐strength sources around the isocenter axis to emulate the fixed‐source static delivery with ∼200 sources.^5^, ^6^, ^7^ One such system, the Akesis Galaxy RTi, is a rotating gamma‐source system containing only 30 ^60^Co sources. Reducing the number of sources from 200 to 30 has the potential to lower the cost of the system, and shorten the downtime for source (re)loading.

The general dosimetric and mechanical aspects of rotating gamma systems have been characterized on stylized head phantoms in previous works,^6^, ^7^ including clinical treatment plan comparisons with a rotating system and a robotic stereotactic radiosurgery system.^8^ However, a dosimetric plan quality comparison between rotating systems and the newest Gamma Knife Icon system has yet to be explored. Additionally, much of the previous testing has been completed on previous versions of rotating gamma systems, excluding the Akesis Galaxy RTi, which has recently received FDA approval and varies in source configuration from previously tested systems. This dosimetric study retrospectively reutilizes patient‐specific images, contours, and dose distributions from patients previously treated with GK at our institution. The resultant dose distributions for the AK are examined alongside the dose distributions for the GK using target dose statistics and plan quality indices in order to provide a meaningful benchmark of the AK performance.

## 2 MATERIALS AND METHODS

### 2.1 The Akesis Galaxy RTi system

**FIGURE 1:**
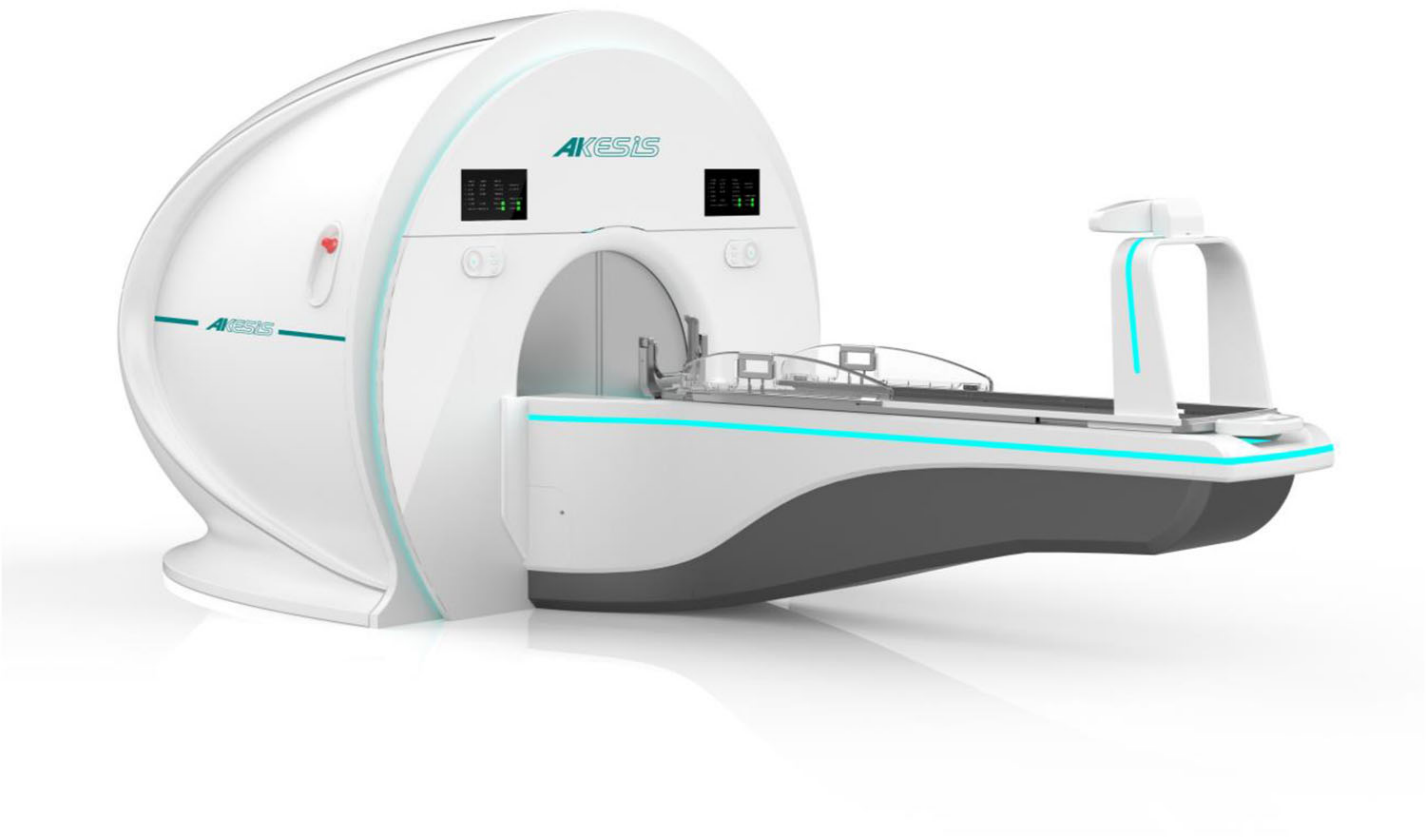
The Akesis Galaxy RTi

The Akesis Galaxy RTi (AK) is a rotating gamma system containing 30 ^60^Co sources in a housing “tray” arranged to produce 30 non‐overlapping, non‐coplanar arcs while rotating at up to 1.5 revolutions per minute (RPM).^7^ The automated collimation system rotates simultaneously with the tray during treatment, and collimates each source to the same aperture size of 4, 8, 14, or 18 mm diameter (as measured at the isocenter). The system requires 8 s to switch between collimator sizes. All 30 sources are loaded into the tray, which is wholly exchanged with a newly‐loaded tray during source reloading. At (re)loading, the system has a dose rate of ≥ 3 Gy/min at isocenter using the largest aperture (∼6000 Ci of ^60^Co).^7^ The system is shown in Figure 1.

The patient can be docked on the table with a gamma angle (i.e. angle between patient head frame and couch) of 70, 90, and 110 degrees to better accommodate lesions situated toward the periphery of the brain. The overall positioning and radiological accuracy of the system is 0.5 mm.^7^ Finally, the source‐to‐axis distance (SAD), or the distance from the individual source to the isocenter, is 388 mm.

### 2.2 The Gamma Knife Icon system

**FIGURE 2:**
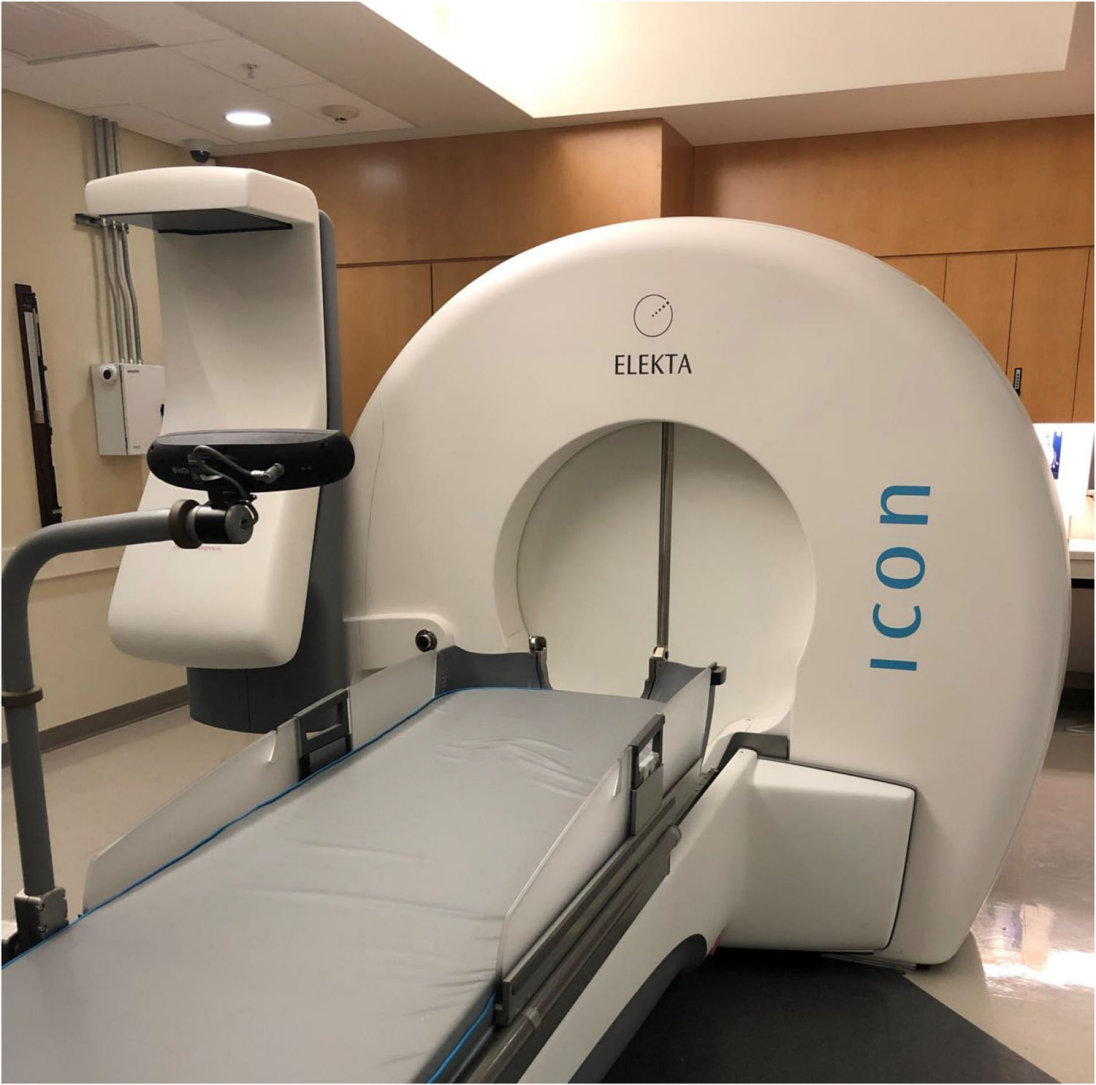
Gamma Knife Icon

The Leksell GK Icon model contains 192 ^60^Co sources. Sources are divided into eight separate sectors of 24 sources, and separated into 5 rings. Each sector can be individually collimated to 4 mm, 8 mm, 16 mm, or blocked, and can switch between collimator sizes within 1 s.^9^ At (re)loading, the system contains 6000–6600 Ci of ^60^Co and has a dose rate of ≥ 3 Gy/min.^10^ The system is shown in Figure 2.

The system can accommodate gamma angles of 70, 90, and 110 degrees, with positional and radiological accuracy each better than 0.5 mm.^9^, ^11^ All beams intersect at the machine isocenter, which is between 374 and 433 mm SAD, depending on the fixed source location within the gantry.^9^

### 2.3 Patient cohort

The patient cohort includes 13 previously‐treated GK patients at our institution with varying disease sites and plan complexity. Patient presentations include five metastatic, two schwannoma, two pituitary adenoma, two trigeminal neuralgia (TN), and two arteriovenous malformation (AVM) cases.

Existing patient image sets and contours from GK are anonymized and exported to the AK TPS for re‐planning. Treatment planning at our institution is completed using forward planning. To maintain consistency for the AK system, the shots and collimators were also selected using forward planning. Therefore, the physicist or dosimetrist completing treatment planning selects the shots and collimators to best conform the dose distribution to the various targets. This allows for a direct comparison between both systems. To facilitate a direct dosimetric comparison between the two systems, AK plans utilize the same prescription doses and isodose levels as those of the original GK plans, therefore the AK plans are matched to the clinically‐approved GK plans. In doing this, the prescription dose is held constant and the same isodose level is required to cover the target for both plans. Prescription dose and isodose normalization were based on our institutional policies, which are based on clinical trial and outcomes data. Finally, to determine the achievable performance of AK, all plans were re‐optimized, without the constraint of maintaining the same prescription isodose line as the original GK plans.

### 2.4 Planning comparison

Dosimetric comparisons between treatment machines are carried out using several dosimetric and plan quality indices, including: target dose statistics, Radiation Therapy Oncology Group (RTOG) conformity index (CI), selectivity, Paddick conformity index (PCI), and the gradient index (GI). These plan quality indices are summarized in Table 1 and are calculated based on the prescription irradiation volume (PIV), the target volume (TV), the volume of the target covered by the prescription isodose level (TV_PIV_), and the overall volume covered by half of the prescription isodose (PIV_0.5_). Target dose statistics include the minimum target dose (D_min_), the mean target dose (D_mean_), the dose to 95% of the target (D_95%_), and the volume receiving 150% of the prescribed dose (V_150%_). The data used for this study are available upon request.

**TABLE 1:** Plan quality indices used for plan comparison: Calculation methods and ideal values

**Plan quality index**	**Definition**	**Ideal value**
RTOG conformity index^11^	PIV/TV	1.0
Selectivity^12^	TV_PIV_/PIV	1.0
Paddick conformity index^13^	TV_PIV_^2^/(TV × PIV)	1.0
Gradient index^12^	PIV_0.5_/PIV	<3.0

Abbreviations: PIV, prescription irradiation volume; PIV_0.5_, overall volume covered by half of the prescription isodose; RTOG, Radiation Therapy Oncology Group; TV, target volume; TV_PIV,_ volume of the target covered by the prescription isodose level.

### 2.5 Statistical analysis

To determine the statistical significance of any differences in dosimetric and plan quality indices, paired *t*‐tests were used with an α of 0.05. Any tests reporting *p* values of < 0.05 indicate a statistically significant difference in the particular index being evaluated between GK and AK. Analysis was completed on the number of shots, treatment time, D_min_, D_mean_, D_95%_, V_150%_, CI, PCI, selectivity, and GI.

## 3 RESULTS

### 3.1 Plan comparison and overview

**FIGURE 3:**
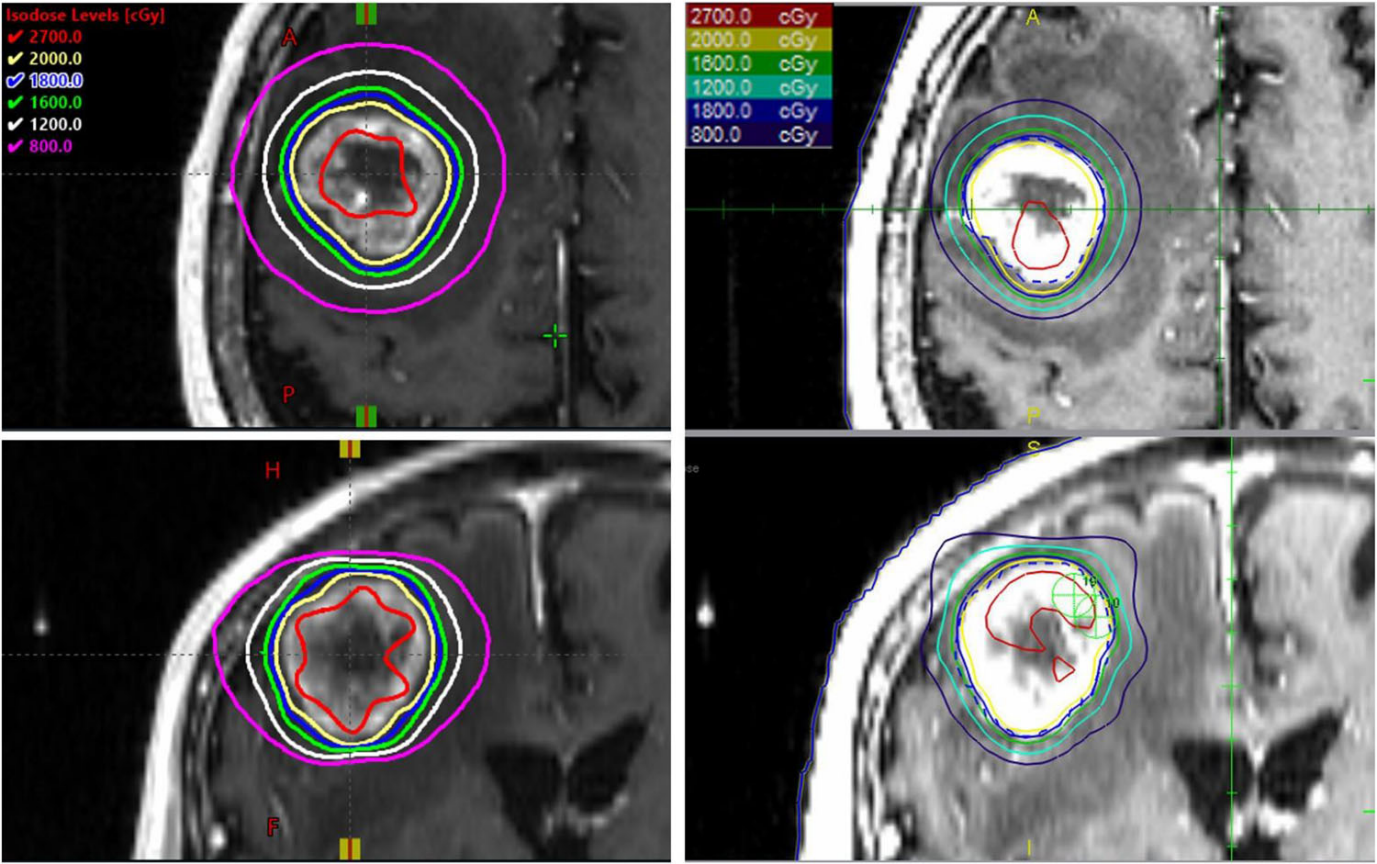
Comparison of isodose lines for a single metastasis patient treated with 18 Gy to the 50% isodose line for GK (left) and AK (right)

The final statistics for plans created for the AK and the GK, including the treatment time, collimator settings used, and number of shots are summarized in Table 2. A comparison of isodose lines for a metastasis patient are illustrated in Figure 3 for the GK and AK. For both systems, delivery time refers to the total beam on time, plus the time needed for collimator switching, rotation (if applicable), and table movement. In this manuscript, treatment time is defined as the sum of the beam‐on times. The beam‐on times for each machine were scaled to a dose rate of 3 Gy/min to ensure that the same source strength was used for the comparisons. In this study, the treatment times for both AK and GK refer only to the time needed to deliver all shots, and do not include collimator switching time or table travel time. The AK plans are prescribed to the same dose and isodose level as the original GK plans. In general, the AK uses fewer shots (*p* = 0.045) and larger available collimator settings. As a result, among the 13 patient plans evaluated, treatment times for 11 patients are shorter for the AK compared to the GK (*p* = 0.047); for one AVM case treatment time is reduced by 88.6 min (62%). However, for two metastatic patients (AK0001 and AK0005), the GK did produce faster plans by up to 8.7 min (27%).

**TABLE 2:** Summary of plan characteristics for the two systems including the system used, disease type, the prescribed dose and isodose line, the treatment time, collimator settings used, and number of shots

**Patient ID**	**Disease type**	**System**	**Prescription**	**Treatment time [min]**	**# Shots (collimation [mm])**
AK0001	Metastatic	GK	18 Gy to 50%	37.7	34 (16,8)
		AK		52.3	16 (14,8,4)
AK0002	Metastatic	GK	20 Gy to 50%	21.9	4 (16,8)
		AK		21.8	3 (14,8,4)
AK0003	Trigeminal neuralgia	GK	80 Gy to 100%	34.6	1 (4)
		AK		30.3	1 (4)
AK0004	Schwannoma	GK	12 Gy to 50%	22.4	3 (4,B)
		AK		19.6	2 (4)
AK0005	Metastatic	GK	22 Gy to 60%	16.7	3 (8,4)
		AK		22.8	5 (8,4)
AK0006	Schwannoma	GK	13 Gy to 60%	17.9	2 (4,B)
		AK		11.0	3 (4)
AK0007	AVM	GK	16 Gy to 50%	143.2	41 (16,8,4)
		AK		54.8	29 (18,14,8,4)
AK0009	Trigeminal neuralgia	GK	80 Gy to 100%	36.6	1 (4)
		AK		23.2	1 (4)
AK0010	AVM	GK	20 Gy to 50%	52.3	12 (4,B)
		AK		22.4	4 (8,4)
AK0011	Pituitary adenoma	GK	18 Gy to 50%	108.9	22 (8,4,B)
		AK		36.1	18 (18,14,8,4)
AK0012	Pituitary adenoma	GK	18 Gy to 50%	37.7	7 (8,4,B)
		AK		17.3	7 (8,4)
AK0014	Metastatic	GK	16–20 Gy to 50%–60%	96.7	33 (16,8,4,B)
		AK		82.1	26 (18,14,8,4)
AK0015	Metastatic	GK	22 Gy to 50%	21.3	3 (16,8)
		AK		15.5	3 (18,8)
*p*‐value				0.047	0.045

The *p*‐value indicates the statistical significance of the result, with *p* < 0.05 indicating a statistically significant difference between AK and GK.

Abbreviations: AK, Akesis Galaxy RTi; AVM, arteriovenous malformation; B, blocked shot; GK, Gamma Knife Icon.

Our institution does not create contours as part of the clinical treatment of trigeminal neuralgia. Because of this, these cases have been excluded from any statistical analysis requiring delineation of the target volume (such as target dose statistics and plan quality indices). For analysis, the remaining patients are sorted into two categories: “Simple” and “Complex”, with the characteristics of each category outlined in the following subsections.

### 3.2 Plan statistics: Simple cases

Patient cases are classified as “Simple” for targets that are small (<2 cc), round, and are located >2.5 cm from any organs‐at‐risk (i.e. optics, brainstem, cochlea). About half of the cohort (5/13) was classified as Simple. Brain metastasis(es) cases can generally be classified as Simple. Target dose statistics and plan quality indices for the Simple cases are shown in Table 3.

**TABLE 3:** Target dose statistics and plan quality indices for patients with simple cases

**Patient ID**	**System**	**Target vol [cc]**	**D_min_ [Gy]**	**D_mean_ [Gy]**	**D_95%_ [Gy]**	**V_150%_** **[cc]**	**V_4Gy_** **[cc]**	**V_12Gy_** **[cc]**	**RTOG CI**	***S***	**PCI**	**GI**
AK0001	GK	13.30	19.0	26.1	21.0	5.14	150.68	20.39	1.37	0.731	0.730	2.77
	AK		16.6	26.4	20.2	5.88	89.86	9.76	1.21	0.826	0.828	2.37
AK0002	GK	1.16	21.0	28.7	23.0	0.50	24.29	3.83	2.03	0.494	0.490	2.80
	AK		20.1	27.8	23.1	0.23	22.66	2.74	1.67	0.600	0.598	2.74
AK0005	GK	0.27	24.3	30.4	26.8	0.02	4.49	0.70	1.61	0.621	0.620	2.66
	AK		22.3	29.3	24.3	0.03	5.93	0.79	1.45	0.688	0.684	3.20
AK0014	GK						302.39	31.53				
	AK						303.36	31.82				
Met #1	GK	10.10	20.7	28.8	23.2	3.95			1.47	0.678	0.680	–
	AK		19.8	30.9	24.5	6.13			1.55	0.645	0.645	–
Met #2	GK	7.51	16.0	21.5	17.4	11.59			1.32	0.759	0.760	–
	AK		12.5	23.9	19.1	14.92			1.55	0.624	0.639	–
Met #3	GK	0.03	25.9	30.7	28.5	3.95			6.06	0.165	0.160	–
	AK		21.0	24.4	25.3	6.13			2.50	0.400	0.400	–
Met #4	GK	0.06	24.3	30.3	27.1	3.95			4.60	0.218	0.220	–
	AK		24.3	27.0	29.2	6.13			2.70	0.370	0.370	–
AK0015	GK	1.71	25.1	34.2	27.6	0.82	32.18	4.71	1.71	0.586	0.590	2.49
	AK		23.4	33.3	29.2	0.88	41.54	4.73	1.61	0.622	0.622	2.63
*p*‐value			0.008	0.369	0.960	0.028	0.473	0.343	0.155	0.146	0.132	0.798

GI is not calculated for multi‐lesion plans. The *p*‐value indicates the statistical significance of the result, with *p* < 0.05 indicating a statistically significant difference between AK and GK.

Abbreviations: AK, Akesis Galaxy RTi; CI, conformity index; GI, gradient index; GK, Gamma Knife Icon; PCI, Paddick conformity index; RTOG, Radiation Therapy Oncology Group; S, selectivity.

For the majority of metastatic cases in this cohort, GK delivers higher D_min_ (16.0–25.9 Gy) and higher D_mean_ (21.5–34.2 Gy) compared to AK (12.5–24.3 Gy and 23.9–33.3 Gy, respectively). However, only the differences in D_min_ were statistically significant, with *p* = 0.008. AK tends to have lower D_min_, higher D_95%_ (19.1–29.2 Gy vs. 17.4–28.5 Gy for GK)_,_ higher V_150%_ (0.03–14.92 cc vs. 0.02–11.59 cc for GK), and larger low dose irradiation volumes (V_4Gy_ 6.91–368.47 cc vs. 4.76–320.09 cc for GK). Only the differences in V150% produced a statistically significant results with *p* = 0.028. The lower D_min_ and higher V_150%_ both imply more dose heterogeneity within the target for AK plans. With regards to plan quality indices, AK tends to give closer to ideal results for the RTOG CI, selectivity, and PCI suggesting that the AK plans are more conformal to the target. However, none of the differences in these indices proved to be statistically significant.

### 3.3 Plan statistics: Complex cases

Patient cases classified as Complex are those containing targets which are large, non‐spherical and/or in close proximity to critical structures (optic nerve or brainstem). AVMs, schwannomas, and pituitary adenomas are often classified as Complex cases. Target dose statistics and plan quality indices for these cases are presented in Table 4.

**TABLE 4:** Target dose statistics and plan quality indices for complex cases

**Patient ID**	**System**	**Target Vol [cc]**	**D_min_ [Gy]**	**D_mean_ [Gy]**	**D_95%_ [Gy]**	**V_150%_ [cc]**	**V_4Gy_** **[cc]**	**V_12Gy_** **[cc]**	**RTOG CI**	***S***	**PCI**	**GI**
AK0004	GK	0.079	12.0	17.3	13.2	0.02	0.57	0.02	1.52	0.658	0.660	3.36
	AK		14.5	18.6	14.6	0.06	1.37	0.11	2.53	0.395	0.420	3.47
AK0006	GK	0.031	13.1	18.4	15.2	–	0.53	0.04	2.58	0.388	0.390	–
	AK		14.8	18.6	15.7	–	0.47	0.05	2.87	0.348	0.350	3.43
AK0007	GK	13.46	9.4	20.6	15.9	1.73	113.63	11.81	1.17	–	–	2.81
	AK		9.4	20.8	16.4	1.91	132.88	17.85	1.39	–	–	2.91
AK0010	GK	0.27	16.1	24.5	24.5	0.15	6.32	1.12	2.41	–	–	2.64
	AK		20.6	22.2	22.2	0.09	18.28	2.94	3.26	–	–	3.45
AK0011	GK	1.95	17.6	25.8	20.4	0.77	23.11	3.28	1.49	0.670	0.670	2.66
	AK		18.1	25.8	21.2	0.78	54.75	7.69	2.50	0.401	0.401	3.11
AK0012	GK	0.505	17.5	27.8	21.1	0.28	5.68	0.77	1.46	0.686	0.690	2.60
	AK		18.1	25.8	20.0	0.20	8.01	0.95	1.48	0.677	0.677	3.04
*p*‐value			0.062	0.485	0.955	0.716	0.086	0.103	0.025	0.130	0.124	0.044

*S* and PCI values are undefined for AK0007 and AK0010 because target volumes are not contoured. The *p*‐value indicates the statistical significance of the result, with *p* < 0.05 indicating a statistically significant difference between AK and GK.

Abbreviations: AK, Akesis Galaxy RTi; CI, conformity index; GI, gradient index; GK, Gamma Knife Icon; PCI, Paddick conformity index; RTOG, Radiation Therapy Oncology Group; S, selectivity.

For the complex cases evaluated, AK delivers higher D_min_ (9.4–20.6 Gy) and higher D_95%_ (14.6–22.2 Gy) compared to GK (9.4–17.6 Gy and 13.2–24.5 Gy, respectively), as well as larger low dose volumes (V_4Gy_ 0.47–132.88 cc vs. 0.57–113.63 cc for GK). The differences in these indices were not found to be statistically significant. While AK provides higher doses, GK provides better selectivity, PI, PCI, and GI for the cases evaluated by a factor of 1.04–1.67. Differences in CI and GI were found to be statistically significant between the two systems, with *p* values of 0.025 and 0.044, respectively. This suggests that, for Complex cases, the GK delivers more conformal dose distributions with sharper gradients compared to AK.

Contours of OARs were completed for three patients (AK0006, AK0011, and AK0012), and OAR doses were compared between GK and AK. These results are illustrated in Table 5. With this limited cohort, GK appears to provide improved dose sparing to the OARs evaluated, with maximum doses for left and right optic nerves below 8 Gy. AK plans illustrate higher optic nerve doses, with maximum doses exceeding 9.5 Gy. AK did provide slightly lower maximum dose for the cochlea contour in AK0006, with a maximum dose of 2.9 Gy, compared to the 3.6 Gy achieved in the GK plan.

**TABLE 5:** Comparison of OAR doses between GK and AK for patients with contoured OARs

**Patient**	**OAR**	**Machine**	**D_min_**	**D_max_**	**D_mean_**
AK0006	Cochlea	GK	25.1	364.8	107.6
		AK	0.7	295.3	104.2
AK0011	Lt optic nerve	GK	66.9	704.1	309.5
		AK	10.4	1791.3	639.3
AK0011	Rt optic nerve	GK	75.9	551.0	225.0
		AK	2.9	1374.7	400.2
AK0012	Lt optic nerve	GK	17.6	607.5	164.9
		AK	0.0	953.9	212.1
AK0012	Rt optic nerve	GK	26.1	756.5	168.6
		AK	0.4	1157.2	283.6

### 3.4 Plan statistics: AK optimized dose distributions

The results presented in Section III.2–III.3 are based on AK plans which are purposely matched to the same prescription isodose level as the original GK plan to facilitate comparison. Five patients (AK0001–AK0005) are re‐optimized, without the prescription isodose level constraint, for the AK system. The results for these re‐optimized plans and the original GK plans are shown in Table 6, with dose statistics and plan quality indices listed in Table 7.

**TABLE 6:** Comparison of re‐optimized AK plans to those planned for GK

**Patient ID**	**System**	**Prescription**	**Treatment time [min]**	**# Shots (collimation [mm])**
AK0001	GK	18.0 Gy to 50%	37.7	34 (16,8)
	AK	18,0 Gy to 50%	46.4	16 (14,8,4)
	AK_op_	22.8 Gy to 64.5%	28.0	10 (18,14,8)
AK0002	GK	20.0 Gy to 50%	21.9	4 (16,8)
	AK	20.0 Gy to 50%	19.2	3 (14,8,4)
	AK_op_	24.9 Gy to 64%	17.6	10 (18,14,8)
AK0003	GK	80.0 Gy to 100%	34.6	1 (4)
	AK	80.0 Gy to 100%	30.2	1 (4)
	AK_op_	76.6 Gy to 100%	30.6	1 (4)
AK0004	GK	12.0 Gy to 50%	22.4	3 (4,B)
	AK	12.0 Gy to 50%	19.5	2 (4)
	AK_op_	15.9 Gy to 78.6%	8.0	2 (4)
AK0005	GK	22.0 Gy to 60%	16.7	3 (8,4)
	AK	22.0 Gy to 60%	22.9	5 (8,4)
	AK_op_	28.5 Gy to 79.7%	9.3	1 (8)

Abbreviations: AK, Akesis Galaxy RTi; AK_op_, re‐optimized Akesis plans; B, blocked shot; GK, Gamma Knife Icon.

**TABLE 7:** Dose statistics and plan quality indices for re‐optimized AK plans

**Patient ID**	**System**	**D_min_ [Gy]**	**D_mean_ [Gy]**	**D_95%_ [Gy]**	**V_150%_ [cc]**	**RTOG CI**	***S***	**PCI**	**GI**
AK0001	GK	19.0	26.1	21.0	5.14	1.37	0.731	0.730	2.77
	AK	16.6	26.4	20.2	5.88	1.21	0.826	0.828	2.37
	AK_op_	19.2	27.8	23.0	0.10	1.03	0.924	0.896	2.98
AK0002	GK	21.0	28.7	23.0	0.50	2.03	0.494	0.490	2.80
	AK	20.1	27.8	23.1	0.23	1.67	0.600	0.598	2.74
	AK_op_	22.1	39.0	24.4	0.10	1.12	0.847	0.771	3.30
AK0004	GK	12.0	17.3	13.2	0.02	1.52	0.658	0.660	3.36
	AK	14.5	18.6	14.6	0.06	2.53	0.395	0.420	3.47
	AK_op_	15.9	18.3	15.9	0.00	1.13	0.789	0.922	2.12
AK0005	GK	24.3	30.4	26.8	0.02	1.61	0.621	0.620	2.66
	AK	22.3	29.3	24.3	0.03	1.45	0.688	0.684	3.20
	AK_op_	25.7	30.9	27.0	0.00	1.15	0.777	0.954	3.97

AK0003 is not included in this table because these targets are not contoured for trigeminal neuralgia cases.

Abbreviations: AK, Akesis Galaxy RTi; AK_op_, re‐optimized Akesis plans; CI, conformity index; GI, gradient index; GK, Gamma Knife Icon; PCI, Paddick conformity index; RTOG, Radiation Therapy Oncology Group; S, selectivity.

In general, fewer shots and larger collimator settings are used in the AK plans, relative to the GK plans. With respect to the original AK plans, the re‐optimized (AK_op_) plans have higher D_min_ (15.9–25.7 Gy), D_mean_ (18.3–39.0 Gy), and D_95%_ (15.9–27.0 Gy) compared to GK (12.0–24.3 Gy, 17.3–30.4 Gy, and 13.2–26.8 Gy, respectively), as well as lower V_150%_ (0.00–0.10 vs. 0.02–5.14 cc). The re‐optimized plans also offer better RTOG CI, selectivity, and PCI compared to GK. AK, AK_op_, and GK have similar GI, which suggests that neither machine offers significantly better dose falloff compared to the other even after re‐optimization.

## 4 DISCUSSION

It is unclear what combination of dosimetric characteristics or plan quality indices are most important. Indeed, this concept of an “ideal” plan may actually be different between patients. For example, a patient with highly hypoxic cells may benefit from higher prescription doses (increased D_min_, D_mean_) with a trade‐off for less conformity.^12^ For these patients, heterogeneity within the plan is also thought to be a positive feature, as higher doses in the center of the tumor are thought to be more effective at controlling hypoxic and radioresitant portions of the tumor, leading to better outcomes.^12^ On the other hand, the strategy for a patient with a target near a critical structure such as the optics may be to accept lower dose to the target in exchange for extremely tight margins on the target (improved RTOG CI, PCI).^12^, ^13^ Therefore, this study is designed to compare the general differences in a large number of common metrics between the AK and the GK.

### 4.1 Treatment times

In our cohort, the AK system consistently produces shorter treatment times than the GK for Complex plans, in one case by as much as 88.6 minutes (62%). In addition to practical concerns stemming from protracted delivery, the shorter treatment times may actually yield a radiobiological advantage, as suggested by Kann *et al.* ^14^

Both the AK and the GK are initially calibrated to an output factor of 1.000 for the largest collimator size available (18 mm for AK and 16 mm for GK). The dose rates for the smaller collimators are scaled based on relative output with respect to the largest collimator. Ultimately, a smaller shot size takes more time to deliver the same dose than a larger shot size because a larger proportion of the beam is blocked. Because of this, relative output factors for the AK for different collimator sizes are 1.000 (18 mm), 0.986 (14 mm), 0.980 (8 mm), and 0.837 (4 mm), while relative output factors for the GK used in this study are 1.000 (16 mm), 0.900 (8 mm), and 0.814 (4 mm).

While both the largest collimators have the same relative output factor of 1, the AK assigns this same dose rate to a larger volume than the GK, which increases its overall delivery efficiency. Further, the actual dose rates for the 4 and 8 mm shots are higher with the AK collimators than those of the corresponding GK collimators, which further increases the delivery efficiency.

For the majority of the plans evaluated, AK uses fewer shots which—in conjunction with the increased delivery efficiency per shot—leads to substantial time savings for treatment delivery. For one case (AK0007), the AK uses 29 shots compared to 41 for the GK, and, more importantly, avoids using as many 4 mm shots, which have much lower output factors and can protract treatment time.

For two patients, AK0001 and AK0005, GK is observed to deliver faster plans compared to AK. In the plan for AK0001, GK uses double the number of shots compared to AK. However, GK uses only the 16 and 8 mm collimators, whereas the AK also uses 8 mm, and 4 mm collimators. Even though substantially fewer shots are used in the AK plan, the use of the larger collimators in the GK plan leads to a total treatment time of 37.7 min compared to the AK plan with a total treatment time of 46.4 min. In the plan for AK0005, both plans utilize 8 and 4 mm collimators, however the AK plan uses four 4 mm shots and one 8 mm shot, while the GK plan uses two 4 mm shots and one 8 mm shot. This leads to a total treatment time for the AK of 22.9 min, compared to 16.7 min for the GK.

### 4.2 Dose statistics for target

Our study indicates that for Simple cases, GK plans have higher D_min_ (*p* = 0.008) and D_mean_ (*p* = 0.369) than AK, while AK plans have higher D_95%_ (*p* = 0.960) and V_150%_ (*p* = 0.028). On the other hand, for the Complex cases, an almost reversed trend is observed: AK delivers higher D_min_ (*p* = 0.062), D_mean_ (*p* = 0.485), and D_95%_ (*p* = 0.955) than GK, with similar V_150%_ (*p* = 0.716). Additionally, for all but one case, AK was observed to deliver larger low dose volumes compared to GK (V_4Gy_, *p* = 0.846). Contouring is not completed at our institution for trigeminal neuralgia cases, therefore these cases are disqualified from analysis of target dose statistics.

The larger low dose volume in AK plans may have several causes. The dosimetric accuracy of the GK profiles has been characterized, demonstrating that physical measurements with GK agree well with the treatment planning system and that field sizes and penumbra are accurately modeled.^15^ Field sizes (full width at half maximum) and the 80%–20% penumbra values of the 8 and 4 mm collimators for the AK were compared to the GK, as shown in Table 8. This table illustrates some differences between the two systems. While the GK tends to have smaller field size dimensions and penumbra in the Z direction (into‐out of the machine, perpendicular to the source rings/arcs), AK provides smaller field dimensions and penumbra in X and Y (parallel to the source rings/arcs). A reduction of the penumbra in the X and Y dimensions will not necessarily reduce the low dose volume of normal tissue irradiated, as beams are entering the tissue in these directions. However, there are no sources oriented along the Z axis, therefore, a small field size and penumbra in this direction will help to spare normal tissue.

**TABLE 8:** Comparison of full width at half maximum field size and 80%–20% penumbra for the 8 and 4 mm collimators of the GK and AK

**Parameter**	**Machine**	**8 mm collimator**			**4 mm collimator**		
		***X***	***Y***	***Z***	***X***	***Y***	***Z***
FWHM	GK	11.0	11.0	9.6	6.1	6.2	5.0
	AK	11.6	11.5	11.5	5.0	4.9	5.0
80%–20%	GK	4.3	4.3	2.6	3.2	3.3	1.8
	AK	3.7	3.0	3.7	2.0	1.9	2.0

Besides differences in field size and penumbra, these two machines also differ in the number of sources and source orientation. For the AK, 30 sources rotate around the patient, creating 30 non‐coplanar arcs. For the GK, the 192 sources are oriented in 5 arcs around the patient. Fewer arcs leads to fewer beam entry points, leading to less areas of normal brain tissue irradiated by low doses. Additionally, since the AK sources are rotating around the patient, beam can be entering from any angle along these 30 arcs, which can lead to a much larger volume of normal tissue irradiation compared to the 192 fixed sources in the GK.

While higher doses within the target can theoretically produce higher levels of control,^12^ previous investigators have suggested that D_min_ may ultimately dictate the overall tumor control probability,^16^, ^17^ though this has not necessarily been demonstrated clinically.^18^ Further analysis and larger cohorts would be needed to determine the effect this could have on clinical treatment efficacy and long‐term survival. Therefore, while these preliminary results do show general trends favoring GK for Simple cases and AK for Complex cases, there may not be a clearly superior approach based on target dose statistics alone.

### 4.3 Re‐optimized AK plans

To facilitate direct comparison between the two systems, the prescription isodose level for the new AK plans are originally assigned the prescription isodose level from the original GK plans. However, it is later demonstrated in our investigation that dose metrics for many AK plans can be further improved with no constraint on the prescription isodose level. Results from five patients (AK0001–AK0005) are presented using typical AK prescription isodose levels (50%–85%) rather than the standard GK prescription isodose level (50%).^8^ After re‐optimizing using a different isodose line, the AK_opt_ plan optimizations have further improved D_min_, D_mean_, and D_95%;_ but lower V_150%_.

### 4.4 Rotating versus fixed‐source system

Clinical outcomes data for intracranial SRS are based on treatments delivered with fixed‐source ^60^Co machines, such as the GK. These machines, while proven to be very effective,^2^, ^3^, ^4^ do have several practical disadvantages in addition to the potential radiobiological effects mentioned earlier. The large number of ^60^Co sources in the unit (192–201) can incur high costs for source replacement. Additionally, GK patients must be postponed during the 3–4‐week time period required for machine breakdown, rigging, source loading, and testing.

Rotating systems like the AK can alleviate some of these issues. Because sources are rotated around the patient, fewer sources are used while still maintaining similar source coverage and treatment angles compared to the GK. Fewer sources decreases the overall production cost of materials, and also allows all sources to be housed in a single tray which can be completely removed and replaced during source exchange.^7^ This leads to significantly less downtime during source exchange compared to GK (∼2 weeks vs. ∼1 month) and lower source exchange costs (∼$500 000 compared to ∼$1 000 000). Finally, the AK offers shorter treatment times due to the larger relative output factors. This shorter treatment time, coupled with the similar performance and plan quality compared to the GK noted in this study, suggests potential advantages for rotating systems. However, one must also consider potential differences in down time between the two systems due to mechanical wear‐and‐tear with the rotating sources, and uncertainties related to source rotation, neither of which were quantified in this study.

## 5 CONCLUSIONS

This study benchmarks the dosimetric performance of the rotating‐source stereotactic AK system against the conventional fixed‐source GK system for a small cohort of previously‐treated GK patients. The AK system offers better conformity and dose falloff for the metastatic cases in this cohort, but also exhibits larger dose heterogeneity compared to the GK system. On the other hand, for the non‐metastatic cases in this cohort, the GK system offers better conformity and dose falloff, with the AK offering higher doses. The results from this limited cohort suggest that the AK can produce largely similar dose distributions and comparable dose metrics as the GK.

## AUTHOR CONTRIBUTION

Emily Draeger: Performed data acquisition and analysis and drafted the manuscript. Zhe (Jay) Chen: Contributed to design of study and review of analysis and manuscript. James E. Hansen: Contributed to design of study and review of analysis and manuscript. Veronica Chiang: Contributed to design of study and review of analysis and manuscript. Christopher J. Tien: Contributed to design of study, performed data analysis, and reviewed manuscript.

## CONFLICTS OF INTEREST

The authors have no conflicts of interest to report.
